# Structure of mouse IP-10, a chemokine

**DOI:** 10.1107/S0907444908007026

**Published:** 2008-05-14

**Authors:** Talat Jabeen, Philip Leonard, Haryati Jamaluddin, K. Ravi Acharya

**Affiliations:** a Department of Biology and Biochemistry, University of Bath, Claverton Down, Bath BA2 7AY, England

**Keywords:** interferon-γ-inducible protein, chemokines

## Abstract

Interferon-γ-inducible protein (IP-10) belongs to the CXC class of chemokines and plays a significant role in the patho­physiology of various immune and inflammatory responses. It is also a potent angiostatic factor with antifibrotic properties. The biological activities of IP-10 are exerted by interactions with the G-protein-coupled receptor CXCR3 expressed on Th1 lymphocytes. IP-10 thus forms an attractive target for structure-based rational drug design of anti-inflammatory molecules. The crystal structure of mouse IP-­10 has been determined and reveals a novel tetrameric association. In the tetramer, two conventional CXC chemokine dimers are associated through their N-terminal regions to form a 12-­stranded elongated β-sheet of ∼90 Å in length. This association differs significantly from the previously studied tetramers of human IP-10, platelet factor 4 and neutrophil-activating peptide-2. In addition, heparin- and receptor-binding residues were mapped on the surface of IP-­10 tetramer. Two heparin-binding sites were observed on the surface and were present at the interface of each of the two β-­sheet dimers. The structure supports the formation of higher order oligomers of IP-10, as observed in recent *in vivo* studies with mouse IP-10, which will have functional relevance.

## Abstract 2

The structure of mouse IP-10 shows a novel tetrameric association.

## 1. Introduction

Chemokines are involved in chemotaxis and activation of leukocytes in immune and inflammatory responses by interacting with their specific G-protein-coupled receptors (Moser & Loetscher, 2001 ▶) and have been divided into C, CC, CXC and CX_3_C subfamilies on the basis of their N-terminal cysteine positions (Zlotnik & Yoshie, 2000 ▶; Ottonello, 2006 ▶).

Interferon-γ-inducible protein (IP-10; CXCL10; 10 kDa) belongs to the CXC family of chemokines and is secreted by a variety of cell types (Baggiolini *et al.*, 1997 ▶). IP-10 acts as an immunoinflammatory mediator in Th1-type inflammatory diseases (Papadakis *et al.*, 2004 ▶), rheumatoid arthritis (Ruschpler *et al.*, 2003 ▶), cardiac allograft rejection (Zhao *et al.*, 2002 ▶), multiple sclerosis (Sorensen, 2004 ▶), atherosclerosis (Heller *et al.*, 2006 ▶), sarcoidosis (Sugiyama *et al.*, 2006 ▶) and prostate cancer (Nagpal *et al.*, 2006 ▶). The receptor for IP-10 has been recognized as CXCR3 (Loetscher *et al.*, 1996 ▶), which is predominantly expressed on activated T lymphocytes (Th1; Sallusto *et al.*, 1998 ▶) in addition to other cell types including NK cells, dendritic cells, macrophages and B cells (Loetscher *et al.*, 1998 ▶; Qin *et al.*, 1998 ▶). Two other physiological ligands for CXCR3 are monokine induced by interferon-γ (Mig; CXCL9; Loetscher *et al.*, 1996 ▶) and interferon-inducible T-cell α chemoattractant (I-TAC; CXCL11; Cole *et al.*, 1998 ▶).

As a consequence of their biological and therapeutic significance, the three-dimensional structures of about 30 chemokines have been studied to date. More recently, the structures of thymus and activation-regulated chemokine (TARC; Asojo *et al.*, 2003 ▶) from the CC chemokines and I-­TAC (Booth *et al.*, 2004 ▶) and stroma cell-derived factor-1α (SDF-1α; Gozansky *et al.*, 2005 ▶) from the CXC chemokines have been determined. The structure of human IP-10 has been determined previously in three different crystal forms (Swaminathan *et al.*, 2003 ▶). Each of these structures formed a distinct tetrameric assembly.

Glycosaminoglycans (GAGs) are linear, highly sulfated and heterogeneous polysaccharides that are often covalently linked to core proteins called proteoglycans that are present on the membrane of cells or within the extracellular matrix. They have been demonstrated to be required for the biological function of chemokines (Yu *et al.*, 2005 ▶) and have been shown to facilitate their oligomerization (Vivès *et al.*, 2002 ▶). Binding of chemokines to the GAG chains of cell-surface proteo­glycans is thought to facilitate the formation of highly local­ized concentrations of chemokines, which in turn provides directional signals for leukocyte migration. Heparan sulfate has been demonstrated to be required for the presentation of chemokines on endothelial cells as well as for *in vivo* recruitment of leukocytes (Wang *et al.*, 2005 ▶).

The physiological relevance of oligomerization is still not clear, but it has intrigued researchers to carry out studies to understand the mechanism of the oligomerization-induced functions of chemokines. *In vivo* studies with monomeric mutants of the CC chemokines RANTES (regulated upon activation, normal T-cell expressed and secreted), macrophage inflammatory protein-1 β (MIP-1 β) and monocyte chemo­attractant protein-1 (MCP-1) showed reduced recruitment of leukocytes (Proudfoot *et al.*, 2003 ▶). Chemokines in monomeric forms have also been shown to be cleared more rapidly *in vivo*, suggesting a role of oligomerization in the localized retention of chemokines (Frevert *et al.*, 2002 ▶). More recent studies on IP-10 mutants have clearly demonstrated the mechanism of oligomerization-dependent recruitment of activated CD8^+^ T cells into mice airways. The monomeric mutants were unable to induce the recruitment, although they showed significant receptor and heparin binding at higher concentrations *in vitro*. This suggests that the oligomerization of IP-10 is essential for its *in vivo* activity (Campanella *et al.*, 2006 ▶).

Various biochemical studies and the recent discovery of its *in vivo* oligomerization-dependent functions prompted us to determine the three-dimensional structure of mouse IP-10. Here, we present the crystal structure of mouse IP-10 at 2.5 Å resolution, which shows a novel oligomeric association. The present results provide an insight into the structural basis of oligomerization and the physiological functions of IP-10 that may contribute to further understanding of the structure and function of chemokines.

## 2. Experimental procedures

### 2.1. Cloning, expression and purification

Mouse IP-10 was cloned and expressed as described by Campanella *et al.* (2003 ▶) with some modifications. The recombinant plasmid was used to transform *Escherichia coli* BL21 (DE3) pLys strain and the culture was grown at 310 K. Protein expression was induced with 0.2 m*M* isopropyl β-d-1-thiogalactopyranoside. Cells were harvested 4 h after induction, pelleted and resuspended in lysis buffer containing 50 m*M* Tris–HCl pH 8.0 and 2 m*M* Na EDTA. Cells were lysed by sonication and the cell lysate was pelleted, resuspended and re-sonicated. 0.01% Triton X-100 was added to the cell suspension to wash away the membrane. The pellet collected after centrifugation at 40 000*g* was dissolved in solubilization buffer containing 7 *M* guanidine–HCl, 0.1 *M* Tris–HCl pH 8.0, 0.15 *M* reduced glutathione and 2 m*M* EDTA. Refolding was per­formed at 277 K following the procedure of Holloway *et al.* (2001 ▶). Refolded protein was diluted fivefold with MilliQ water and applied onto an SP-Sepharose column (Fast Flow, GE Healthcare) pre-equilibrated with 50 m*M* Tris–HCl, 50 m*M* NaCl pH 8.0. The bound fractions were eluted using a 0.05–0.75 *M* gradient of NaCl in 50 m*M* Tris–HCl buffer pH 8.0 at a flow rate of 1 ml min^−1^. Protein quantification was performed using the BCA assay (Pierce). Fractions containing mouse IP-­10 were identified by SDS–PAGE and loaded onto a C4 RP-HPLC column pre-equilibrated with 0.1% trifluoro­acetic acid in HPLC-grade water. The protein was eluted with a linear gradient of acetonitrile containing 0.08% trifluoro­acetic acid at a flow rate of 1 ml min^−1^. The effluent was monitored at 230 nm. Fractions containing a single band of mouse IP-10, as identified by SDS–PAGE, were pooled and lyophilized.

### 2.2. Crystallization, data collection and processing

Pure lyophilized protein was dissolved in 50 m*M* Tris–HCl pH 8.0 to a concentration of 10 mg ml^−1^ and crystallization was performed by the hanging-drop vapour-diffusion method. Initial screening produced thin plates, which were optimized to improve the crystal quality. Diffraction-quality crystals were finally obtained in 0.1 *M* Tris–HCl pH 8.0, 0.2 *M* CaCl_2_ and 35% PEG 3350 after three weeks at 289 K. A data set was collected to 2.5 Å resolution at the Synchrotron Radiation Source (Station PX14.2), Daresbury, UK and was processed and scaled using the programs *DENZO* and *SCALEPACK* (Otwinowski & Minor, 1997 ▶). The crystals belong to space group *C*2, with unit-cell parameters *a* = 109.9, *b* = 71.5, *c* = 39.6 Å, β = 110.0°. The complete data statistics are given in Table 1 ▶.

### 2.3. Structure determination and refinement

The structure of mouse IP-10 was solved by maximum-likelihood molecular replacement using the program *Phaser* (Read, 2001 ▶) from the *CCP*4 program suite (Collaborative Computational Project, Number 4, 1994 ▶). The coordinates of one molecule of human IP-10 in a truncated form (residues 9–­65; PDB code 1o7y; Swaminathan *et al.*, 2003 ▶) were used to build the initial search model and a clear solution was obtained in space group *C*2 with four molecules in the asymmetric unit. A stacking arrangement of molecules in the unit cell for this solution was observed in *Coot* (Emsley & Cowtan, 2004 ▶), which yielded no unfavourable intermolecular contacts. Initial cycles of refinement resulted in an *R*_cryst_ of 36.0% and an *R*_free_ of 42.9%. Iterative cycles of energy minimization, individual *B*-factor refinement and simulated annealing were carried out in *CNS* (Brünger *et al.*, 1998 ▶), alternated with model building using *Coot* (Emsley & Cowtan, 2004 ▶). Residues were replaced according to the amino-acid sequence of mouse IP-10. The positions of 81 water molecules were identified from the |*F*_o_| − |*F*_c_| electron-density maps above 3σ and were checked manually for their interactions with protein atoms. The missing residues at the N- and C-termini were added as their density appeared with progressive refinement. Refinement was stopped when no further improvement in *R*_free_ was made. The final model has an *R*_cryst_ of 27.6% and an *R*_free_ of 30.3%. Crystallographic statistics are given in Table 1 ▶. Figures were produced using the programs *PyMOL* (DeLano Scientific LLC; http://www.pymol.org) and *POV-Ray* (http://www.povray.org).

## 3. Results and discussion

### 3.1. Quality of the structure

The structure of mouse IP-10 has been determined at 2.5 Å resolution. The final model contains four molecules in the asymmetric unit: *A* (residues 1–68), *B* (residues 1–67), *C* (residues 4–68) and *D* (residues 4–67). Electron density for the first three N-terminal residues could not be observed in molecules *C* and *D*, whereas density for C-terminal residues beyond 67 (in molecules *B* and *D*) and 68 (in molecules *A* and *C*) could not be observed. Some disorder was observed at the C-terminal ends, thus affecting the length of the C-terminal helices. However, the C-terminal residues were not found to interact directly with heparin and CXCR3 (Campanella *et al.*, 2003 ▶) and hence the absence of these residues did not affect our analysis. There are no breaks in the main chain and most of the side chains are located in density. Some residues at the surface show disorder or more than one conformation. The structure has good geometry, with root-mean-square (r.m.s.) deviations of 0.010 Å and 1.4° in bond lengths and bond angles, respectively. The Ramachandran plot (Ramachandran & Sasisekharan, 1968 ▶) obtained using *PROCHECK* (Las­kowski *et al.*, 1993 ▶) showed that 77.8% of residues fall in most favoured regions, whereas 19.6% and 2.6% were in additionally and generously allowed regions, respectively. The refinement statistics are given in Table 1 ▶.

### 3.2. The mouse IP-10 structure

**Figure 1:**
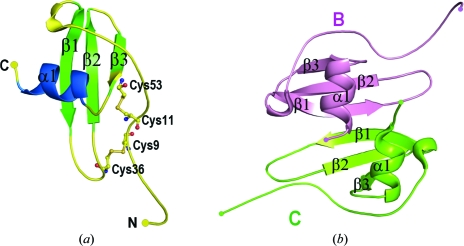
(*a*) Monomeric structure of mouse IP-10. The three β-strands and α-­helices are labelled. The N- and C-termini are indicated. The disulfide bonds stabilizing the N-terminal extended loop are shown in ball-and-stick representation. (*b*) Ribbon diagram of the pseudosymmetrical dimer formed between molecules *B* (pink) and *C* (green), consisting of a six-stranded β-sheet with two antiparallel α-helices. Molecules *A* and *D* form a similar dimer.

The structure of mouse IP-10 contains four molecules in the asymmetric unit. Each molecule exhibits the typical chemokine structural fold consisting of an extended N-­terminal loop, three antiparallel β-strands and a C-terminal helix lying obliquely across the β-sheet (Fig. 1 ▶ *a*). All the molecules have a similar core structure, with differences in the N- and C-termini and loop regions. Superposition of the C^α^ atoms of the four molecules shows an r.m.s. deviation of 0.5–1.07 Å. In each molecule, the N-terminal extended loop is stabilized by two disulfide bonds between conserved cysteines (9:36 and 11:53). A dimer is formed by molecules *A* and *D*, which are related by pseudosymmetry. Molecules *B* and *C* form a similar pseudosymmetrical dimer (Fig. 1 ▶ *b*). In each of the two dimers, the β1 strands from each monomer associate to form a six-stranded β-sheet with two antiparallel α-helices lying on one face of the β-sheet. This structural organization is similar to the dimeric CXC chemokine structures. The two dimers have an r.m.s.d of 0.59 Å (122 C^α^ atoms). The C^α^ backbones of the pseudosymmetrical dimers in the present structure deviate by an average of 1.17 Å (r.m.s.) from human IP-10 dimers (PDB codes 1o7y, 1o7z, 1o80; Swaminathan *et al.*, 2003 ▶), while they show a C^α^ r.m.s. deviation of 1.4–1.6 Å compared with the dimers formed by interleukin-8 (IL-8; PDB code 3il8; Baldwin *et al.*, 1991 ▶), platelet factor 4 (PF4; PDB code 1rhp; Zhang *et al.*, 1994 ▶), neutrophil-activating peptide-2 (NAP-2; PDB code 1nap; Malkowski *et al.*, 1995 ▶), growth-related oncogene-β (Groβ; PDB code 1qnk; Qian *et al.*, 1999 ▶) and SDF-1α (PDB code 1a15; Gozansky *et al.*, 2005 ▶) from the CXC family.

**Figure 2:**
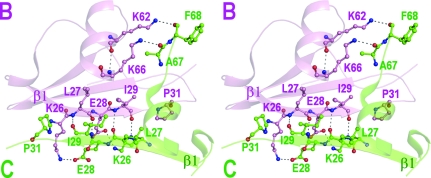
Stereoview of the intermolecular interactions at the *B*–*C* interface. Residues from molecules *B* and *C* are shown in pink and green, respectively. Similar interactions between the β1 strands were observed at the *A*–*D* interface.

The dimers (*A*–*D* and *B*–*C*) are primarily stabilized by reciprocal interactions between opposing β1 strands. The main-chain hydrogen bonding at the interface is limited to reciprocal interactions between residues Leu27 and Ile29 (thus accounting for four hydrogen bonds) owing to the presence of Pro31, which disrupts the β-structure. Similar interactions were observed in human IP-10 dimers owing to the conserved Pro31. However, in other CXC chemokines this position is occupied by residues other than Pro and hence four residues are involved in main-chain reciprocal interactions compared with two in the IP-10 structures. Additional interactions at the interface are contributed by salt bridges between residues Lys26 and Glu28 of the β1 strands from both the molecules in addition to extensive van der Waals contacts. The involvement of the C-terminal helices in the intermolecular interactions is limited owing to their short lengths and consists of two hydrogen bonds between residues Lys62 N^ζ^ and Lys66 N^ζ^ of molecule *B* with Phe68 O and Ala67 O of molecule *C*, respectively. The ionic interactions at the C-terminal regions are absent in molecules *A* and *D* and only van der Waals contacts are made (Table 2 ▶, Fig. 2 ▶). The interfaces between *A* and *D* and between *B* and *C* bury 1280 and 1170 Å^2^ of solvent-accessible surface area, respectively, which is comparable to the buried surface areas of other CXC chemokine dimers.

### 3.3. Mouse IP-10 tetramer

**Figure 3:**
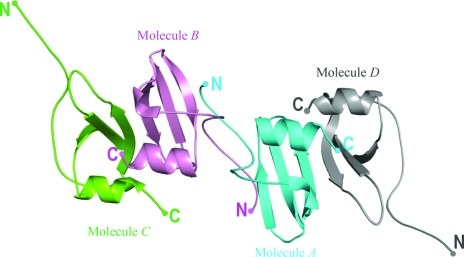
Schematic representation of the mouse IP-10 structure. The tetramer is formed by the association of dimers *B*–*C* (shown in pink and green) and *A*–*D* (shown in blue and grey).

**Figure 4:**
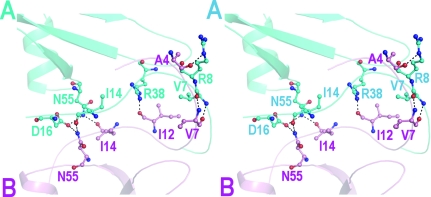
Stereo representation of the hydrogen bonds formed at the *A*–*B* interface. Residues from molecule *A* are shown in blue and those from molecule *B* in pink.

In addition to the *A*–*D* and *B*–*C* dimers, another dimer is formed between molecules *A* and *B* in the mouse IP-10 structure. This results in a distinct tetrameric assembly that is formed by the association of two pseudosymmetrical dimers: *A*–*D* and *B*–*C* (Fig. 3 ▶). The tetramer has an elongated structure with approximate dimensions of 90 × 40 Å^2^. Intermolecular contacts in the tetramer are through molecules *A* and *B*. The two molecules associate through their N-terminal loops in an antiparallel fashion such that their α-helices lie on one face of the β-strands, while the interacting N-terminal loops are on the back of the strands. The rotation axis parallel to the β-sheets and dissecting the *A*–*B* dimer relates chains *A* and *B* with a rotation angle of 179.1°, as calculated by *LSQMAN* (Kleywegt & Jones, 1994 ▶). This is a novel association in which the tetramer consists of two six-stranded antiparallel β-sheets, with an antiparallel sheet formed by the N-­terminal regions between the two six-stranded β-­sheets and all four helices lying on one face of the β-sheet (Fig. 4 ▶). This type of association differs significantly from many of the chemokine oligomeric structures studied to date. Intermolecular association through N-terminal regions has been observed previously in CC chemokines such as MIP-1β (Lodi *et al.*, 1994 ▶) and RANTES (Shaw *et al.*, 2004 ▶). However, these dimeric structures differ considerably from the *A*–*B* dimer in the present structure. In these CC chemokines, two monomers associate together such that their three-stranded β-sheets face each other with a short β-sheet formed by the interacting N-­terminal regions in the centre. The C-terminal helix of each of the monomer lies on the exterior face of the β-sheet.

The *A*–*B* dimer is primarily stabilized by the reciprocal main-chain hydrogen bonds between the two N-terminal loops. However, the extended N-terminal loop of one molecule also interacts with the 30s loop and 50s loop of other molecule in a reciprocal manner such that a total of seven hydrogen bonds are formed at the *A*–*B* interface (Fig. 4 ▶, Table 2 ▶). In addition to these hydrogen bonds, the two molecules have an extensive network of hydrophobic interactions. The solvent-accessible area at the interface in the dimer is reduced by 1770 Å^2^, which is higher than the buried surface area of the pseudosymmetrical dimers *A*–*D* and *B*–*C* (1280 and 1170 Å^2^, respectively).

**Figure 5:**
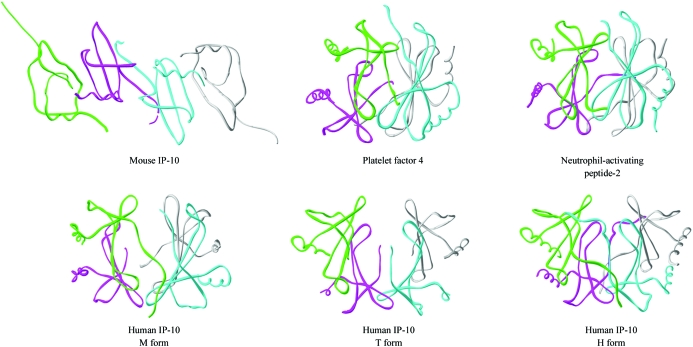
Backbone tracing of the oligomeric structures formed in mouse and human IP-10, platelet factor 4 and neutrophil-activating peptide-2. Four chains are shown in each structure, in which a typical chemokine dimer is formed between the green and magenta chains and the blue and grey chains, respectively. Note the different association of dimers in each structure. This figure was drawn using the program *SwissPDBViewer* (Guex & Peitsch, 1997 ▶).

The mouse IP-10 structure differs significantly in its tetrameric association from human IP-10 structures. The human IP-­10 tetramer in the monoclinic space group (M form) is formed by the association of two pseudosymmetrical dimers such that the two six-stranded β-sheets face each other while the C-terminal helices are present on the exterior. This arrangement of subunits is similar to the tetramers of the CXC chemokines PF4 (Zhang *et al.*, 1994 ▶) and NAP-2 (Malkowski *et al.*, 1995 ▶) (Fig. 5 ▶). In the tetragonal (T form) and hexagonal (H form) space groups, the human IP-10 dimers associate with the symmetry-related dimers through their β3 strands to form a 12-stranded antiparallel β-sheet structure that has a sharp kink in the middle which gives an open barrel-shaped structure to the complex. However, in the H-form tetramer, the dimers also form N-terminal asymmetric interactions, thus bringing the chains closer (Swaminathan *et al.*, 2003 ▶). The crystal packing of the human IP-10 structures in all three space groups show different arrangements of molecules compared with the mouse IP-10 structure. In contrast to the mouse IP-10 structure, which has an elongated cylindrical shape, all these tetramers form globular-shaped structures (Fig. 5 ▶).

### 3.4. Glycosaminoglycan-binding regions

**Figure 6:**
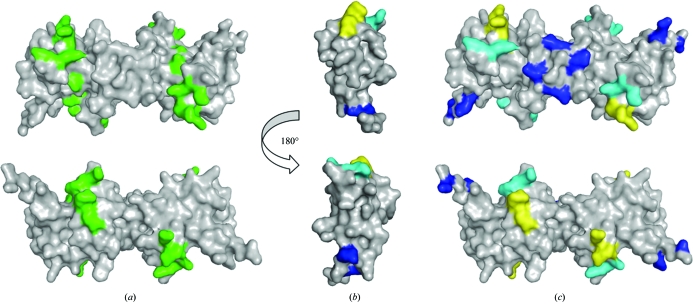
(*a*) Surface representation of the heparin-binding residues (shown in green) in the mouse IP-10 structure. The views are related by a 180° rotation about the *y* axis (the lower view is identical to that in Fig. 3 ▶). (*b*) The receptor-binding regions on the surface of the monomeric form of IP-10. Arg5 and Arg8 are shown in blue, Arg20, Arg22 and Ile24 in yellow and Lys46 and Lys47 in cyan. Two views are shown that are related by a rotation of 180° about the *y* axis. (*c*) The mapping of receptor-binding regions on the mouse IP-10 tetramer. The two views are related by a 180° rotation about the *y* axis (the lower view is the same as that in Fig. 3 ▶).

Glycosaminoglycans (GAGs) on cells bearing chemokine receptors have been reported to facilitate chemokine–receptor interactions (Hoogewerf *et al.*, 1997 ▶). The heparin-binding residues of mouse IP-10 were determined by side-directed mutagenesis. The mutational analysis indicated residues Arg20, Arg22, Ile24, Lys26, Lys46 and Lys47 to be potentially involved in heparin binding (Campanella *et al.*, 2003 ▶). Arg22 and Lys46 were found to be critical for binding; these residues are conserved and also form the heparin-binding site in human IP-10 (Swaminathan *et al.*, 2003 ▶) and PF4 (Mayo *et al.*, 1995 ▶). Single- and double-point mutations in the C-terminal helix did not affect heparin binding. However, the mutation of four basic residues at the C-terminus to neutral and acidic residues resulted in reduced heparin binding (Campanella *et al.*, 2003 ▶). The reduced heparin binding arising from these mutations is likely to be the consequence of an electrostatic effect (Campanella *et al.*, 2003 ▶). The heparin-binding residues are present at the N-­loop/β1 strand and β2 strand/40s loop junctions in the IP-10 structure. In the pseudosymmetrical dimer *A*–*D* (or *B*–*C*), these residues co-localize to form a basic cluster that lines a groove that is present on the dimeric interface along the β1 strands. Association of the two pseudosymmetrical dimers, with each dimer having one binding site, results in the presence of two putative heparin-binding sites per tetramer. Both binding sites lie on one face of the tetramer, opposite to the helices, running along the β1 strands and extending across the edges, thus representing maximal possible interactions with the heparin (Fig. 6 ▶ *a*).

The potential heparin-binding residues in human IP-10 were identified as Arg22, Lys46, Lys47, Lys48, Lys62 and Lys66 (Swaminathan *et al.*, 2003 ▶), which partially overlap with the mouse IP-10 heparin-binding site. In the human IP-10 (M form) and PF4 structures, the heparin-binding residues form a ring around the tetramers, which follows a scattered and convoluted path in the tetramers of the T and H forms of human IP-10. In all these structures, the C-terminal helices are involved in the dimeric interface such that the binding site runs perpendicular to the C-terminal helices, with residues from the loop connecting the N-terminal region and the β1 strand forming the extended edges. Hence, the association of the dimers might form two binding sites per tetramer, similar to those observed in mouse IP-10. Heparin–chemokine structural modelling studies indeed showed two heparin oligosaccharides docked onto the PF4 tetramer, each oligosaccharide adopting a curved shape that runs across the pseudosymmetrical dimer perpendicular to the α-helices and bridges the two dimers (Lortat-Jacob *et al.*, 2002 ▶). However, the previously modelled SDF-1α–heparin complex shows the oligosaccharide binding to the dimer along the interface between the β1 strands, with the α-helices lying on the opposite face (Sadir *et al.*, 2001 ▶). The oligosaccharide thus adopts a straight and extended shape. More recently, the crystal structure of SDF-1α in complex with heparin disaccharide revealed two binding sites, one of which is present at the dimer interface, thus supporting the previous studies (Murphy *et al.*, 2007 ▶). The mouse IP-10 structure analysis indicates that the heparin oligosaccharide may bind to the IP-­10 dimer in a similar fashion to that observed in SDF-1α. GAGs have been shown to induce chemokine oligomerization, thus forming a chemokine gradient (Proudfoot, 2006 ▶). In addition to chemokine presentation to the receptors, GAGs may play an active role in the chemokine function. For example, RANTES in complex with GAGs has been shown to have anti-HIV activity (Burns *et al.*, 1999 ▶). Further studies are required to understand the modulation of chemokine activity by GAGs.

### 3.5. CXCR3-binding regions

IP-10-induced physiological functions are mediated by the binding of IP-10 to cell-surface CXCR3. Mutagenesis studies of mouse IP-10 identified the residues that are involved in binding to the receptor (Campanella *et al.*, 2003 ▶). Residues in the 20s loop (Arg20, Arg22), β1 strand (Ile24) and 40s loop (Lys46 and Lys47) were found to be important for receptor binding. The N-terminal residues preceding the first cysteine, specifically Arg5 and Arg8, were found to be involved but not critical for receptor binding. However, Arg8 was found to be critical for CXCR3-mediated signalling. Arg8 and Lys46 are conserved in the human and mouse CXCR3 ligands. Single and double mutations in the C-terminal helix did not affect receptor binding (Campanella *et al.*, 2003 ▶). The CXCR3-binding site partially overlapped with the heparin-binding site, which further indicates that heparin may play an active role in the biological function of mouse IP-10. Fig. 6 ▶(*b*) shows the regions involved in receptor binding in the IP-­10 monomeric structure. The receptor-binding regions are preserved when mapped onto the surface of the tetramer (Fig. 6 ▶ *c*), indicating that the oligomerization of IP-10 may not affect its receptor binding. In human IP-10, immunological studies indicated residues 20–36 to be involved in CXCR3 binding, which also overlaps with the receptor-binding residues of mouse IP-10. Oligomerization has been demonstrated to be an essential requirement for the biological function of IP-10 (Campanella *et al.*, 2006 ▶). Cytokine receptors have been reported to undergo dimerization upon cytokine binding (Rodriguez-Frade *et al.*, 1999 ▶). The CXC chemokine SDF1-α has been reported to induce dimerization of the CXCR4 receptor upon binding (Vila-Coro *et al.*, 1999 ▶). Receptor clustering is known to occur during the initiation of ligand-induced internalization in triggering the biological responses. However, receptor clustering has not yet been reported for CXCR3 molecules.

### 3.6. Biological significance of oligomerization

IP-10 has been shown to exist as higher order oligomeric forms under physiological conditions (Campanella *et al.*, 2006 ▶). However, *N*-methylated Leu27 monomeric mutants of IP-10 had reduced heparin and CXCR3 binding but were able to induce CXCR3 internalization and chemotaxis of CD8^+^ T cells expressing CXCR3 at tenfold higher concentrations than wild-type IP-10 *in vitro*. However, the monomeric mutants failed to induce *in vivo* recruitment of activated CD8^+^ T cells. Oligomerization, rather than heparin and CXCR3 binding, was found to be essential for *in vivo* recruitment of T cells (Campanella *et al.*, 2006 ▶). In the IP-10 structure, Leu27 is present in the β1 strand and is involved in reciprocal main-chain hydrogen bonds with Ile29 of the other monomer forming the antiparallel dimer *A*–*D* (or *B*–*C*). The presence of the *N*-methyl group disrupts this interaction and thus prevents the formation of dimers and possibly higher order complexes. Only oligomeric forms of IP-10 were able to bind to endothelial and epithelial cells in a GAG-dependent manner. The binding of oligomeric IP-10 to endothelial cells was shown to be required for the transendothelial migration of CXCR3-expressing lymphocytes. The binding creates a haptotactic gradient, thus inducing the recruitment of activated T cells (Campanella *et al.*, 2006 ▶). Oligomerization is therefore important for the activity of IP-10.

## 4. Conclusion

The mouse IP-10 structure presents a novel tetramer in which two typical CXC chemokine dimers associate through their N-­terminal regions to form a tetrameric assembly. Moreover, the free N-terminal regions of two molecules at opposite ends of the tetramer increase the possibility of further association of molecules to form higher order oligomers. The presence of multiple heparin-binding sites on IP-10 oligomers might play a role in the structural stabilization of oligomers together with a role in the binding of oligomers to endothelial cells to induce the recruitment of CXCR3-expressing T cells. The cell-bound GAGs may also induce IP-10 oligomerization on the cell surface. The present study contributes to the existence of IP-10 in different oligomeric forms which is important for its *in vivo* activity. Both mouse and human IP-10 form similar dimers, interacting though their β1 strands, as observed for other CXC chemokines. However, the heparin- and CXCR3-binding sites in human and mouse IP-10 only partially overlap. Both the structures present two heparin-binding sites per tetramer and the receptor-binding sites are preserved on the tetrameric surfaces. Investigation of the physiological significance of oligomerization is currently under way. In addition, structural studies of IP-10 in complex with GAGs will be required in order to differentiate GAG-mediated and GAG-independent oligomerization and their functional relevance. Since mutational studies indicated that the C-terminal helix was not directly involved in binding to both heparin and CXCR3, structural studies of IP-10 in complex with heparin and CXCR3 are required in order to establish the role of the C-terminal helix in binding and its importance in chemokine function.

## Supplementary Material

PDB reference: mouse IP-10, 2r3z, r2r3zsf

## Figures and Tables

**Table 1:** X-ray data-collection, processing and refinement statistics Values in parentheses are for the highest resolution shell.

PDB code	2r3z
Space group	*C*2
Unit-cell parameters (Å, °)	*a* = 109.9, *b* = 71.5, *c* = 39.6, β = 110.0
Resolution range (Å)	50.0–2.50 (2.57–2.50)
Total No. of measured reflections	25840
No. of unique reflections	9824
Completeness (%)	98.2 (89.7)
*V*_M_ (Å^3^ Da^−1^)	2.1
No. of molecules in the ASU	4
*R*_merge_† (%)	9.1 (30.4)
*I*/σ(*I*)	6.9 (3.2)
*R*_cryst_‡ (%)	27.6
*R*_free_§ (%)	30.3
No. of protein atoms	2044
No. of water molecules	81
R.m.s. deviations	
Bond lengths (Å)	0.010
Bond angles (°)	1.4
Mean *B* factors (Å^2^)	
Main-chain atoms	55.5
Side-chain atoms and waters	56.2
All atoms	56.9
Ramachandran plot	
Residues in most favoured regions (%)	77.8
Residues in additionally allowed regions (%)	19.6
Residues in generously allowed regions (%)	2.6

† *R*_merge_ = 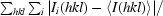 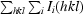, where *I*_*i*_(*hkl*) is the observed intensity of reflection *i* and 〈*I*(*hkl*)〉 is the average intensity of multiple observations.

‡ *R*_cryst_ = 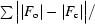 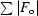, where *F*_o_ and *F*_c_ are the observed and calculated structure-factor amplitudes, respectively.

§ *R*_free_ is equal to *R*_cryst_ for a randomly selected 8% subset of reflections excluded from refinement.

**(a):** *A*–*B* interface.

Molecule *A*	Molecule *B*	Distance (Å)
Val7 N	Val7 O	2.80
Val7 O	Val7 N	2.65
Arg8 N^∊^	Ala4 O	3.11
Ile14 O	Asn55 N^δ2^	2.74
Asp16 O^δ2^	Asn55 N^δ2^	3.14
Arg38 N^∊^	Ile12 O	3.15
Asn55 N^δ2^	Ile14 O	3.09

**(b):** *A*–*D*/*B*–*C* interface.

Molecule *A* (*B*)	Molecule *D* (*C*)	Distance† (Å)
Lys26 N^ζ^	Glu28 O^∊1^	3.35 (2.87)
Leu27 N	Ile29 O	3.35 (3.03)
Leu27 O	Ile29 N	3.12 (2.85)
Glu28 O^∊1^	Lys26 N^ζ^	3.42 (3.27)
Ile29 N	Leu27 O	2.92 (3.05)
Ile29 O	Leu27 N	2.99 (3.10)
Lys62 N^ζ^	Phe68 O	— (3.21)
Lys66 N^ζ^	Ala67 O	— (3.25)

† Values in parentheses are for the *B*–*C* dimer.

## References

[bb1] Asojo, O. A., Boulègue, C., Hoover, D. M., Lu, W. & Lubkowski, J. (2003). *Acta Cryst.* D**59**, 1165–1173. 10.1107/s0907444903009454 12832759

[bb2] Baggiolini, M., Dewald, B. & Moser, B. (1997). *Annu. Rev. Immunol.* **15**, 675–705. 10.1146/annurev.immunol.15.1.675 9143704

[bb3] Baldwin, E. T., Weber, I. T., St Charles, R., Xuan, J. C., Appella, E., Yamada, M., Matsushima, K., Edwards, B. F., Clore, G. M., Gronenborn, A. M. & Wlodawer, A. (1991). *Proc. Natl Acad. Sci. USA*, **88**, 502–506. 10.1073/pnas.88.2.502 PMC50839 1988949

[bb4] Booth, V., Clark-Lewis, I. & Sykes, B. D. (2004). *Protein. Sci.* **13**, 2022–2028. 10.1110/ps.04791404 PMC2279818 15273303

[bb5] Brünger, A. T., Adams, P. D., Clore, G. M., DeLano, W. L., Gros, P., Grosse-Kunstleve, R. W., Jiang, J.-S., Kuszewski, J., Nilges, M., Pannu, N. S., Read, R. J., Rice, L. M., Simonson, T. & Warren, G. L. (1998). *Acta Cryst.* D**54**, 905–921. 10.1107/s0907444998003254 9757107

[bb6] Burns, J. M., Lewis, J. K. & DeVico, A. L. (1999). *Proc. Natl Acad. Sci. USA*, **96**, 14499–14504.

[bb7] Campanella, G. S., Grimm, J., Manice, L. A., Colvin, R. A., Medoff, B. D., Wojtkiewicz, G. R., Weissleder, R. & Luster, A. D. (2006). *J. Immunol.* **177**, 6991–6998. 10.4049/jimmunol.177.10.6991 17082614

[bb8] Campanella, G. S., Lee, E. M., Sun, J. & Luster, A. D. (2003). *J. Biol. Chem.* **278**, 17066–17074. 10.1074/jbc.M212077200 12571234

[bb9] Cole, K. E., Strick, C. A., Paradis, T. J., Ogborne, K. T., Loetscher, M., Gladue, R. P., Lin, W., Boyd, J. G., Moser, B., Wood, D. E., Sahagan, B. G. & Neote, K. (1998). *J. Exp. Med.* **187**, 2009–2021. 10.1084/jem.187.12.2009 PMC2212354 9625760

[bb10] Collaborative Computational Project, Number 4 (1994). *Acta Cryst.* D**50**, 760–763.

[bb11] Emsley, P. & Cowtan, K. (2004). *Acta Cryst.* D**60**, 2126–2132. 10.1107/S0907444904019158 15572765

[bb12] Frevert, C. W., Goodman, R. B., Kinsella, M. G., Kajikawa, O., Ballman, K., Clark-Lewis, I., Proudfoot, A. E., Wells, T. N. & Martin, T. R. (2002). *J. Immunol.* **168**, 3550–3556. 10.4049/jimmunol.168.7.3550 11907118

[bb13] Gozansky, E. K., Louis, J. M., Caffrey, M. & Clore, G. M. (2005). *J. Mol. Biol.* **345**, 651–658. 10.1016/j.jmb.2004.11.003 15588815

[bb14] Guex, N. & Peitsch, M. C. (1997). *Electrophoresis*, **18**, 2714–2723. 10.1002/elps.1150181505 9504803

[bb15] Heller, E. A., Liu, E., Tager, A. M., Yuan, Q., Lin, A. Y., Ahluwalia, N., Jones, K., Koehn, S. L., Lok, V. M., Aikawa, E., Moore, K. J., Luster, A. D. & Gerszten, R. E. (2006). *Circulation*, **113**, 2301–2312. 10.1161/CIRCULATIONAHA.105.605121 16682613

[bb16] Holloway, D. E., Hares, M. C., Shapiro, R., Subramanian, V. & Acharya, K. R. (2001). *Protein Exp. Purif.* **22**, 307–317. 10.1006/prep.2001.1434 11437607

[bb17] Hoogewerf, A. J., Kuschert, G. S., Proudfoot, A. E., Borlat, F., Clark-Lewis, I., Power, C. A. & Wells, T. N. (1997). *Biochemistry*, **36**, 13570–13578. 10.1021/bi971125s 9354625

[bb18] Kleywegt, G. J. & Jones, T. A. (1994). *CCP4/ESF–EACBM Newsl. Protein Crystallogr.* **31**, 9–14.

[bb19] Lortat-Jacob, H., Grosdidier, A. & Imberty, A. (2002). *Proc. Natl Acad. Sci. USA*, **99**, 1229–1234. 10.1073/pnas.032497699 PMC122172 11830659

[bb20] Laskowski, R. A., Moss, D. S. & Thornton, J. M. (1993). *J. Mol. Biol.* **231**, 1049–1067. 10.1006/jmbi.1993.1351 8515464

[bb21] Lodi, P. J., Garrett, D. S., Kuszewski, J., Tsang, M. L., Weatherbee, J. A., Leonard, W. J., Gronenborn, A. M. & Clore, G. M. (1994). *Science*, **263**, 1762–1767. 10.1126/science.8134838 8134838

[bb22] Loetscher, M., Gerber, B., Loetscher, P., Jones, S. A., Piali, L., Clark-Lewis, I., Baggiolini, M. & Moser, B. (1996). *J. Exp. Med.* **184**, 963–969. 10.1084/jem.184.3.963 PMC2192763 9064356

[bb23] Loetscher, M., Loetscher, P., Brass, N., Meese, E. & Moser, B. (1998). *Eur. J. Immunol.* **28**, 3696–3705. 10.1002/(SICI)1521-4141(199811)28:11<3696::AID-IMMU3696>3.0.CO;2-W 9842912

[bb24] McDonald, I. K. & Thornton, J. M. (1994). *J. Mol. Biol.* **238**, 777–793. 10.1006/jmbi.1994.1334 8182748

[bb25] Malkowski, M. G., Wu, J. Y., Lazar, J. B., Johnson, P. H. & Edwards, B. F. (1995). *J. Biol. Chem.* **270**, 7077–7087. 10.1074/jbc.270.13.7077 7706245

[bb26] Mayo, K. H., Ilyina, E., Roongta, V., Dundas, M., Joseph, J., Lai, C. K., Maione, T. & Daly, T. J. (1995). *Biochem. J.* **312**, 357–365. 10.1042/bj3120357 PMC1136271 8526843

[bb27] Moser, B. & Loetscher, P. (2001). *Nature Immunol.* **2**, 123–128. 10.1038/84219 11175804

[bb28] Murphy, J. W., Cho, Y., Sachpatzidis, A., Fan, C., Hodsdon, M. E. & Lolis, E. (2007). *J. Biol. Chem.* **282**, 10018–10027. 10.1074/jbc.M608796200 PMC3684283 17264079

[bb29] Nagpal, M. L., Davis, J. & Lin, T. (2006). *Biochem. Biophys. Acta*, **1762**, 811–818. 10.1016/j.bbadis.2006.06.017 16934957

[bb30] Ottonello, L. (2006). *Curr. Drug Targets*, **7**, 1. 10.2174/138945006775270178 16454705

[bb31] Otwinowski, Z. & Minor, W. (1997). *Methods Enzymol.* **276**, 307–326. 10.1016/S0076-6879(97)76066-X 27754618

[bb32] Papadakis, K., Prehn, A. J., Zhu, D., Landers, C., Gaiennie, J., Fleshner, P. R. & Targan, S. (2004). *Inflamm. Bowel Dis.* **10**, 778–788. 10.1097/00054725-200411000-00013 15626897

[bb33] Proudfoot, A. E. (2006). *Biochem. Soc. Trans.* **34**, 422–426. 10.1042/BST0340422 16709177

[bb34] Proudfoot, A. E., Handel, T. M., Johnson, Z., Lau, E. K., LiWang, P., Clark-Lewis, I., Borlat, F., Wells, T. N. & Kosco-Vilbois, H. M. (2003). *Proc. Natl Acad. Sci. USA*, **100**, 1885–1890. 10.1073/pnas.0334864100 PMC149928 12571364

[bb35] Qian, Y. Q., Johanson, K. O. & McDevitt, P. (1999). *J. Mol. Biol.* **294**, 1065–1072. 10.1006/jmbi.1999.3333 10600366

[bb36] Qin, S., Rottman, J. B., Myers, P., Kassam, N., Weinblatt, M., Loetscher, M., Koch, A. E., Moser, B. & Mackay, C. R. (1998). *J. Clin. Invest.* **101**, 746–754. 10.1172/JCI1422 PMC508621 9466968

[bb37] Ramachandran, G. N. & Sasisekharan, V. (1968). *Adv. Protein. Chem.* **23**, 283–438. 10.1016/s0065-3233(08)60402-7 4882249

[bb38] Read, R. J. (2001). *Acta Cryst.* D**57**, 1373–1382. 10.1107/s0907444901012471 11567148

[bb39] Rodriguez-Frade, J. M., Vila-Coro, A. J., de Ana, A. M., Albar, J. P., Martinez, A. C. & Mellado, M. (1999). *Proc. Natl Acad. Sci. USA*, **96**, 3628–3633. 10.1073/pnas.96.7.3628 PMC22345 10097088

[bb40] Ruschpler, P., Lorenz, P., Eichler, W., Koczan, D., Hanel, C., Scholz, R., Melzer, C., Thiesen, H. J. & Stiehl, P. (2003). *Arthritis Res. Ther.* **5**, R241–R252. 10.1186/ar783 PMC193722 12932287

[bb41] Sadir, R., Baleux, F., Grosdidier, A., Imberty, A. & Lortat-Jacob, H. (2001). *J. Biol. Chem.* **276**, 8288–8296. 10.1074/jbc.M008110200 11087743

[bb42] Sallusto, F., Lenig, D., Mackay, C. R. & Lanzavecchia, A. (1998). *J. Exp. Med.* **187**, 875–883. 10.1084/jem.187.6.875 PMC2212187 9500790

[bb43] Shaw, J. P., Johnson, Z., Borlat, F., Zwahlen, C., Kungl, A., Roulin, K., Harrenga, A., Wells, T. N. C. & Proudfoot, A. E. (2004). *Structure*, **12**, 2081–2093. 10.1016/j.str.2004.08.014 15530372

[bb44] Sorensen, T. L. (2004). *Curr. Neurovasc. Res.* **1**, 183–190. 10.2174/1567202043480143 16185193

[bb45] Sugiyama, K., Mukae, H., Ishii, H., Kakugawa, T., Ishimoto, H., Nakayama, S., Shirai, R., Fujii, T., Mizuta, Y. & Kohno, S. (2006). *Respirology*, **11**, 708–714. 10.1111/j.1440-1843.2006.00933.x 17052298

[bb46] Swaminathan, G. J., Holloway, D. E., Colvin, R. A., Campanella, G. K., Papageorgiou, A. C., Luster, A. D. & Acharya, K. R. (2003). *Structure*, **11**, 521–532. 10.1016/s0969-2126(03)00070-4 12737818

[bb47] Vila-Coro, A. J., Rodriguez-Frade, J. M., Martin De Ana, A., Moreno-Ortiz, M. C., Martinez, A. C. & Mellado, M. (1999). *FASEB J.* **13**, 1699–1710. 10506573

[bb48] Vivès, R. R., Sadir, R., Imberty, A., Rencurosi, A. & Lortat-Jacob, H. (2002). *Biochemistry*, **41**, 14779–14789. 10.1021/bi026459i 12475226

[bb49] Wang, L., Fuster, M., Sriramarao, P. & Esko, J. D. (2005). *Nature Immunol.* **6**, 902–910. 10.1038/ni1233 16056228

[bb50] Yu, Y., Sweeney, M. D., Saad, O. M., Crown, S. E., Hsu, A. R., Handel, T. M. & Leary, J. A. (2005). *J. Biol. Chem.* **280**, 32200–32208. 10.1074/jbc.M505738200 16033763

[bb51] Zhang, X., Chen, L., Bancroft, D. P., Lai, C. K. & Maione, T. E. (1994). *Biochemistry*, **33**, 8361–8366. 10.1021/bi00193a025 8031770

[bb52] Zhao, D., Hu, X. Y., Miller, G. G., Luster, A. D., Mitchell, R. N. & Libby, P. (2002). *J. Immunol.* **169**, 1556–1560. 10.4049/jimmunol.169.3.1556 12133984

[bb53] Zlotnik, A. & Yoshie, O. (2000). *Immunity*, **12**, 121–127. 10.1016/s1074-7613(00)80165-x 10714678

